# Cytochrome P450-Dependent Metabolism of Caffeine in *Drosophila melanogaster*

**DOI:** 10.1371/journal.pone.0117328

**Published:** 2015-02-11

**Authors:** Alexandra Coelho, Stephane Fraichard, Gaëlle Le Goff, Philippe Faure, Yves Artur, Jean-François Ferveur, Jean-Marie Heydel

**Affiliations:** 1 CNRS 6265, INRA 1324, Université de Bourgogne, Centre des Sciences du Goût et de l’Alimentation, F-21000, Dijon, France; 2 INRA, CNRS, UNSA, UMR 1355, Institut Sophia Agrobiotech, F-06903, Sophia Antipolis, France; Kansas State University, UNITED STATES

## Abstract

Caffeine (1, 3, 7-trimethylxanthine), an alkaloid produced by plants, has antioxidant and insecticide properties that can affect metabolism and cognition. In vertebrates, the metabolites derived from caffeine have been identified, and their functions have been characterized. However, the metabolites of caffeine in insects remain unknown. Thus, using radiolabelled caffeine, we have identified some of the primary caffeine metabolites produced in the body of *Drosophila melanogaster* males, including theobromine, paraxanthine and theophylline. In contrast to mammals, theobromine was the predominant metabolite (paraxanthine in humans; theophylline in monkeys; 1, 3, 7-trimethyluric acid in rodents). A transcriptomic screen of Drosophila flies exposed to caffeine revealed the coordinated variation of a large set of genes that encode xenobiotic-metabolizing proteins, including several cytochromes P450s (CYPs) that were highly overexpressed. Flies treated with metyrapone—an inhibitor of CYP enzymes—showed dramatically decreased caffeine metabolism, indicating that CYPs are involved in this process. Using interference RNA genetic silencing, we measured the metabolic and transcriptomic effect of three candidate CYPs. Silencing of *CYP6d5* completely abolished theobromine synthesis, whereas *CYP6a8* and *CYP12d1* silencing induced different consequences on metabolism and gene expression. Therefore, we characterized several metabolic products and some enzymes potentially involved in the degradation of caffeine. In conclusion, this pioneer approach to caffeine metabolism in insects opens novel perspectives for the investigation of the physiological effects of caffeine metabolites. It also indicates that caffeine could be used as a biomarker to evaluate CYP phenotypes in *Drosophila* and other insects.

## Introduction

Caffeine (1, 3, 7-trimethylxanthine) is a typical purine alkaloid that is produced in a variety of plants, including coffee (*Coffea arabica*) and tea (*Camellia sinensis*) [1]. Caffeine is involved in plant chemical defense, acting as a repellent, pesticide and allelopathic agent [2–4]. With its psychostimulant, cardiac and diuretic effects, caffeine is one of the most widely used plant secondary metabolites, primarily as a food additive or an ingredient in drugs [5].

Various insect studies have revealed that caffeine can induce similar effects than in vertebrates: inhibition of feeding and sleep [6–8], increased heart rate [9], and mutagenic and mitotic action [10,11]. In insects in particular, caffeine can affect olfactory and visual associative learning, as demonstrated by studies conducted in honeybees [12]. Caffeine can also affect the reproductive (egg laying ability) [3,13,14] and dopaminergic systems, calcium exchange and potassium currents in the central nervous system of insects [15–17]. In Drosophila, the effect of caffeine was measured in regard to bitter taste-induced aversive behavior [18]. Caffeine induces similar repulsive effects to a male sex pheromone, as shown by both the male courtship behavior and the feeding response [19,20]. Several gustatory receptors sensitive to caffeine, such as Gr66a, Gr33a and Gr93a, were also characterized [19,21].

All of these effects may involve metabolites derived from caffeine, which could have, as in humans, their own biological activities [22–24]. Caffeine derivatives in the human liver, metabolized by the cytochrome P450 oxidase enzyme system (in particular by the CYP1A2 isoenzyme), include three major dimethylxanthine metabolites (paraxanthine, theobromine and theophylline) and one hydroxylated metabolite (1, 3, 7-trimethyluric acid) [25]. Paraxanthine is the predominant caffeine-derived metabolite in humans, but 1, 3, 7-trimethyluric acid and theophylline are the major metabolites found in rodents and monkeys, respectively [26–28].

Among the compounds known to induce toxic effects in *Drosophila melanogaster*, caffeine has been studied primarily in regard to its regulatory effect on detoxification enzymes. Exposure to caffeine enabled the identification of CYP enzymes involved in insecticidal or toxic host plant resistance and metabolism [29,30]. These studies led to a better understanding of insecticide resistance [31] and the regulation of CYP expression [32–34]. CYPs have also been proposed to interact in the metabolism of odorant compounds [35,36]. Cytochrome P450s make up a diverse and important gene super-family in all organisms. In insects, CYPs are known to catalyze a diverse range of chemical reactions important for both developmental processes and the detoxification of exogenous compounds. We focused on the *CYP12d1*, *CYP6a8* and *CYP6d5* genes, which showed the largest amplitude of variation in our initial tests. These enzymes were also selected because they belong to two of the four large clades of insect P450 genes: the CYP2 clade, the CYP3 clade (*CYP6a8*, *CYP6d5*), the CYP4 clade and the mitochondrial P450 clade (*CYP12d1*).

Interestingly, there have been few insect reports providing a global picture of the CYP-related metabolism of a xenobiotic compound. Our study takes into account the activity of the genes induced by xenobiotic compounds as well as the catalytic function of the coded enzymes leading to compound degradation. More specifically, we identified the metabolites of caffeine in *Drosophila melanogaster* and screened the “xenobiotic-metabolizing-enzyme genes” affected by caffeine exposure. Among the genes strongly impacted by caffeine exposure, three CYP candidates were silenced, the effects of which were measured on caffeine metabolism together with the coordinated variation of expression between the three CYPs.

## Materials and Methods

### Chemicals

Caffeine (58–08–2), Theobromine (83–67–0), Theophylline (58–55–9), Paraxanthine (611–59–6) and Metyrapone (54–36–4) were purchased from Sigma-Aldrich (St. Louis, MO, USA), and 1, 3, 7 trimethyluric acid (5415–44–1) from ChemCruz (Santa Cruz, CA, USA).

### Drosophila strains and rearing conditions

Flies were reared on a standard yeast/cornmeal/agar medium at 25°C on a 12L:12 D cycle. w^1118^ (y1 w+) and P{Act5C-GAL4}25FO1/CyO strains, identified as w^1118^ and *Actin*-GAL4, respectively, were obtained from the Bloomington stock center. All of the UAS-RNAi strains (ds*CYP12d1* (109256); ds*CYP6a8* (4884); ds*CYP6d5* (12139)) were purchased from the Vienna Drosophila Resource Center (stock ID indicated in parentheses). The UAS RNAi*CYP*/*Actin*-GAL4 system was used to target all tissues (with an *Actin*-GAL4 driver) potentially involved in the metabolism of caffeine and to inhibit (with UAS-RNAi*CYP*) the expression of the selected CYPs in the targeted tissues.

### Caffeine treatment and microarray analysis

Four-day-old adult males were starved for 12 hours in a vial containing a filter paper impregnated with water before being transferred to caffeine-rich medium (18 mM; Sigma-Aldrich) for 12 hours. Food mixed with water served as a control. Taking into account the studies reported by Le Goff G (2006), and Willoughby L, (2006) [29–52], we decided to expose adult male flies for 12 hours on an 18 mM caffeine-rich medium to obtain a trade-off between the optimal induction of CYP genes and the lower mortality for the flies. For each food treatment (caffeine and water), RNA was isolated from 10 fly bodies (thorax and abdomen) using Isol RNA Lysis reagent (5Prime). All samples were prepared in triplicate to permit statistical analysis. Probe labeling, hybridization to single color Agilent 4x44k arrays, scanning and statistical analysis were performed by the IMAXIO company. Genes showing a 2-fold change and a significant p-value < 0.05 were considered to be differentially expressed. Microarray data obtained from this study can be accessed at NCBI GEO (GSE59084).

### RNA extraction and RT-qPCR

Total RNA was extracted from 10 fly bodies using Isol RNA Lysis reagent (5Prime) and treated with RNAse-free DNAse (Euromedex) to avoid genomic DNA contamination. Total RNA (1 μg) was reverse-transcribed using the iScript cDNA Synthesis Kit (BioRad). The qPCR reactions were carried out using a MyIQ (BioRad) and the IQ SYBR Green SuperMix (BioRad). Each reaction was performed in triplicate and all results were normalized to the tubulin and rp-49 mRNA levels and calculated using the ∆∆Ct method.

The following forward and reverse primers were used: *tubulin* TGTCGCGTGTGAAACACTTC and AGCAGTAGAGCTCCCAGCAG; *rp-49* CCCAAGGGTATCGACAACAG and GTTCGATCCGTAACCGATGT; *cyp12d1p* TTAGCTTGTTCATGTGCC and ATTTACGTGGGTCCCGTTC; *cyp6a8* GGCTGAGGTGGAGGAGGT and CGATGACGAAGTTTGGATGA; *cyp6d5* AAGCAACTGCCTGCGAAC and CAATAATGTCGATGGCGTATGT. For each gene, primer efficiency was calculated: *tubulin* (1.90), *rp-49* (2.00), *cyp12d1p* (1.98), *cyp6a8* (1.92); *cyp6d5* (2.00) [37].

### Western-blot

Protein extracts were prepared from male adult flies. Equal amounts of protein were separated on a 4–15% SDS-polyacrylamide gel (Bio-Rad) and blotted using standard procedures. The membrane was incubated with a polyclonal anti-CYP6d5 (1/5000, Proteogenix) and a specific antibody against Actin (1/2000, Abcam) was used for loading controls.

### Caffeine exposure

Four-day-old male adult flies, starved for 12 hours under humid conditions and pre-exposed for 12 hours to a caffeine-rich medium (18 mM), were placed under humid conditions for 3 hours and transferred, without anesthesia, into a MultiCAFE device [38]. Briefly, pre-exposed individuals were transferred into a box containing 4 capillaries filled with a red dye used for further quantification (0.2 mg/mL sulforhodamine, Sigma-Aldrich), 100 mM sucrose (Euromedex) and 0.04 μM radiolabeled caffeine (8–^14^C, Bio trend, 3.7.10^3^ Bq/μL). Flies were fed in the dark for 2 hours at 27°C under high humidity (>60%).

**Fig 1:**
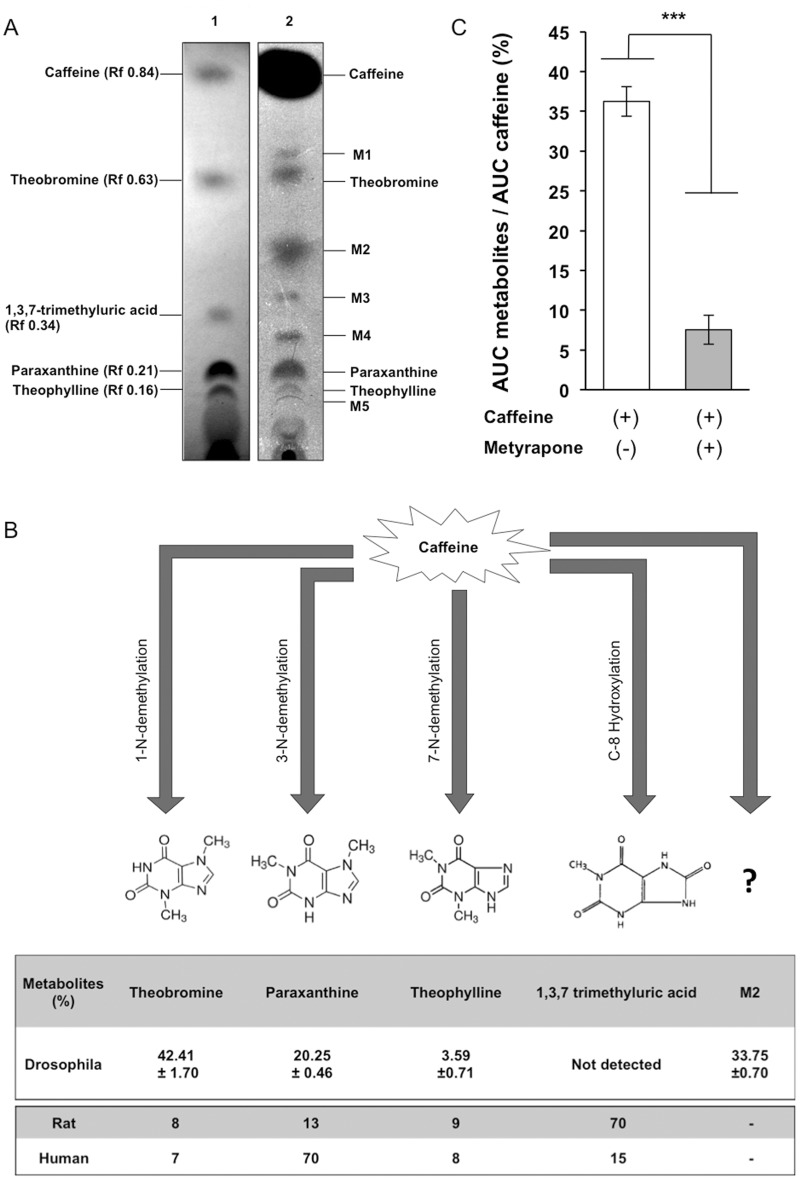
Caffeine metabolism in *Drosophila melanogaster* and the influence of cytochrome P450 inhibitor. A- Separation of non-radiolabeled standards of caffeine and metabolites (lane 1) with a homogenate of wild-type four-days-old *Drosophila* male flies (w^1118^) exposed with radiolabeled caffeine for 2 hours (lane 2) on thin layer chromatography. B- The total amount of caffeine derived metabolites was strongly decreased in the body of male flies after exposure to caffeine mixed with metyrapone (+), as compared to control food (-). The amounts were evaluated by densitometry which is based on the comparison of the areas under the curve (AUC) for both metabolites and caffeine. Bars indicate the mean values (±s.e.m.). Significant differences are indicated by asterisks (Student’s t-test *** p < 0.001; n = 3). C- Interspecific variation for the level of major caffeine metabolites. The proportions of theobromine, paraxanthine and theophylline and of the unidentified M2 metabolite detected in fly bodies after caffeine exposure (18mM) are indicated as the mean (±s.e.m.; n = 4). In *Drosophila melanogaster*, these amounts were evaluated by measuring the relative intensity of the radiolabelled signal associated with each metabolite after thin layer chromatography separation. The proportion of these compounds detected in *Drosophila melanogaster* was compared with that reported for *Rattus norvegicus* and *Homo sapiens* [25–28].

For the metyrapone experiment (Fig. 1B), the 4 capillaries were filled with 100 mM sucrose and 0.04 μM radiolabeled caffeine, as well as 25 mM metyrapone (Sigma-Aldrich).

### Radioactive caffeine and thin layer chromatography (TLC)

We initially performed mass spectrometry analysis to identify caffeine metabolites but faced technical problems, including low metabolism recording and matrix effect on mass detection, which led to a lack of reproducibility (with four attempts). If a similar experiment is never reported in insects, it may be partly because xenobiotic metabolic activity can be inhibited by endogenous compounds present in homogenates [40,41]. This was the reason for the development of a radiolabeled TLC method.

Exposed flies with red-dye-colored abdomens were pooled into groups of 50, homogenized with 150 μL of lysis buffer (Tris 0.1 M, 0.1% SDS, 20% glycerol, 10% protease inhibitor), and centrifuged for 5 min at 16000 g. An aliquot of the supernatant (5 μL) was mixed with scintillation liquid, and the radioactivity was counted with a Tri-Carb 3110TR scintillation counter (PerkinElmer). A similar amount of each sample, or radiolabeled caffeine alone (positive control), was directly applied to a thin layer chromatography plate (Nano-SIL NH2/UV, Macherey-Nagel). The mobile phase used for the separation was composed of chloroform, dichloromethane and isopropanol (v/v/v 4:2:1). After migration, the plate was autoradiographed. The total amount of radiolabeled caffeine-derived metabolites was quantified by densitometry and normalized to non-metabolized caffeine using ImageJ (Software, NIH, Bethesda, MD, USA). Each experiment was conducted in triplicate for appropriate statistical analysis.

### Statistical analyses

For qPCR, transcript level ratios were compared between strains using the Relative Expression Software Tool (REST, REST-MCS beta software version 2) with 2000 iterations [39]. For the radioactivity assay, the amount of caffeine metabolites was compared between control strains and transgenic or treated flies. Normal distribution and homoscedasticity of the variances were checked. In two-group comparisons, Student’s t tests were performed for parametrically distributed data. When more than two groups were compared, ANOVA was performed for parametrically distributed data using R software.

## Results

### Caffeine metabolism

*Drosophila melanogaster* adult males fed with radiolabeled caffeine for 2 hours produced eight caffeine-derived metabolites. These metabolites were distinctly separated by thin layer chromatography (Fig. 1A-lane 2) and additionally identified based on their physical characteristics (retention factors = RFs) compared to commercially available compounds (Fig. 1A-lane 1). The pattern of *Drosophila* caffeine-derived metabolites was compared with that observed in mammals, in which caffeine degradation generally yields four primary metabolites: theobromine, 1, 3, 7-trimethyluric acid, paraxanthine and theophylline (RFs: 0.63, 0.34, 0.21, 0.16, respectively) (Fig. 1A-lane 1).

The pattern of caffeine-derived metabolites in mammals partially overlapped with that obtained in *Drosophila melanogaster*. Notably, theobromine, paraxanthine and theophylline were found in both human and flies. Five additional metabolites detected remain unidentified (M1–M5; Fig. 1A-lane 2). However, the relative amount of caffeine metabolites diverged between *flies*, rat and human (Fig. 1B). While the caffeine ingested by Drosophila was metabolized into 42% theobromine, 20% paraxanthine, and 4% theophylline, mammals produced much less theobromine and slightly more theophylline. Humans also produced more paraxanthine than the two other species. The presence of the M2 metabolite (34%) in Drosophila did not match the mammalian standard metabolite tested. Reciprocally, 1, 3, 7-trimethyluric acid was detected in mammals, but apparently not in Drosophila.

### Genes induced by caffeine exposure

Next, we screened for xenobiotic-metabolizing-enzyme genes (XMEs) induced by caffeine exposure. We carried out a microarray experiment (with a Drosophila pangenomic array on flies exposed or not exposed to caffeine). Interestingly, several genes belonging to all major classes of detoxification enzymes, including XMEs, were among those that were the most highly induced (Tables 1 and 2). Among XME genes, several CYPs were up-regulated (including *CYP12d1*, *CYP6a8* and *CYP6d5*), whereas others were down regulated (Table 1). Remarkably, all induced CYPs were already known to be inducible by a broad range of compounds, such as phenobarbital or the herbicide atrazine (Table 1). In a similar manner, several CYPs known to be repressed by paraquat, a compound known to induce stress by producing reactive oxygen species, were also down-regulated by caffeine [42] (Table 1). Our screen also found several other major detoxification enzymes known to be regulated by different xenobiotics (Table 2).

**Table 1:** Expression of CYP genes in *Drosophila melanogaster* after caffeine exposure.

Gene name	Microarray	P value	Quantitative PCR	P value	Regulation by other compounds ^a^
	Up or down (-) regulation		Up or down (-) regulation		(+) up-expression or
					(-) down-expression
CYP12d1-p	21.41	1.7E-3	15	1.0E-3	+ (phenobarbital, atrazine, piper nigrum, piperonyl butoxide, pyrethrum, ethanol, DDT, chlorpromazine)
CYP12d1-d	19.55	1.5E-3	15	1.0E-3	
CYP6a8	13.48	3.2E-3	20.5	1.0E-3	+ (phenobarbital, piper nigrum, ethanol, DDT, chlorpromazine)
CYP6d5	3.46	2.6E-3	11.8	1.0E-3	+ (phenobarbital, atrazine, piper nigrum, piperonyl butoxide, paraquat, cadmium, ethanol, zinc, rotenone)
CYP4p1	2.95	1.9E-3	2.1	1.0E-2	+(paraquat, tunicamycin, piperonyl butoxide, ethanol, cadmium, rotenone)
CYP304a1	2.85	2.9E-2	2.1	5.0E-2	+(atrazine)
CYP28a5	2.45	3.0E-3	Nd	nd	+(paraquat, tunicamycin, ethanol, rotenone)
CYP12a5	2.42	5.0E-3	3.5	1.0E-2	+(cadmium, ethanol, rotenone)
CYP6a9	2.39	6.3E-3	3.9	1.0E-2	+(ethanol, rotenone)
CYP6a20	2.30	5.0E-3	2.0	1.0E-2	+(cadmium, copper, paraquat)
CYP6w1	2.06	4.2E-3	Nd	nd	+(phenobarbital, atrazine, piper nigrum, piperonyl butoxide, cadmium, zinc, ethanol, paraquat)
CYP313a3	-2.05	4.4E-2	Nd	nd	-
CYP6a18	-2.39	3.8E-3	Nd	nd	-(paraquat)
CYP4d8	-2.55	3.3E-3	Nd	nd	-
CYP316a1	-2.81	3.9E-3	Nd	nd	-
CYP4e1	-3.55	2.2E-3	Nd	nd	-(paraquat), +(tunicamycin)
CYP4d20	-4.20	2.6E-3	-2.3	1.0E-3	-(paraquat, phenobarbital)
CYP313a1	-4.29	3.6E-3	-5.3	1.0E-3	-(paraquat, phenobarbital), + (tunicamycin)
CYP4ac1	-4.95	3.5E-3	-3.5	1.0E-3	+(endosulfan)
CYP4ac2	-9.61	3.5E-3	-3.0	1.0E-3	-(paraquat)
CYP4d21	-15.43	3.5E-3	-9.8	1.0E-3	-(paraquat, phenobarbital)

Adult males were exposed to 18mM caffeine during 12 hours.

^a^ Data extracted from the following references [42], [32], [55], [56], [57], [52], [34], [58], [59], [60] and from RNAseq experiments referenced in modENCODE treatment expression data in Flybase (http://www.flybase.org).

**Table 2:** Expression of major detoxification genes in *Drosophila melanogaster* after caffeine exposure.

Gene name	Up (+) or down (-) regulation	P value	Regulation by other compounds ^(a)^
Esterase			
Alpha-Est7	-2.74	2.2E-3	Rotenone, ethanol, cadmium
Est-6	-3.00	3.1E-3	Paraquat, ethanol, heat shock, zinc
Alpha-Est2	-4.06	2.9E-3	-
Glutathione-S-transferase			
GSTD6	4.05	8.4E-3	Rotenone, ethanol
GSTE1	4.04	3.3E-4	Paraquat, ethanol, heat shock, phenobarbital, cadmium
GSTD5	3.08	6.7E-3	Ethanol, cadmium
GstE12	-2.09	1.1E-4	Heat shock, cadmium, ethanol, rotenone, copper, paraquat
GSTD10	-2.40	2.3E-2	Heat shock, cadmium, ethanol, rotenone, atrazine
GSTE9	-2.46	1.8E-3	paraquat
GSTD8	-2.84	6.8E-3	-
GstZ1	-3.22	1.9E-4	copper
GSTE10	-3.74	2.2E-3	-
GstD11	-4.04	2.5E-3	-
UDP-glycosyltransferase			
CG6475	2.58	1.9E-02	-
CG4302	-2.28	5.5E-04	ethanol
CG6850	-2.59	3.6E-05	-
Ugt35b	-2.86	2.5E-05	-
CG17322	-2.87	1.6E-03	cadmium
CG30438	-3.34	2.4E-04	-
Ugt37b1	-3.43	2.3E-05	-
ATP-binding cassette transporter			
CG8908	3.36	1.7 E-3	-
CG4562	-2.04	8.6 E-3	-
CG31792	-2.09	3.5 E-3	-
CG9664	-2.77	3.4 E-3	-
CG33970	-3.05	1.7 E-3	-

Adult males were exposed to 18mM caffeine during 12 hours.

^(a)^ according to data available from RNAseq experiments referenced in modENCODE treatment expression data in Flybase (http://www.flybase.org), [57], [52].

### Pharmacological and genetic silencing of CYP: effect on caffeine metabolism

Given that the expression of several CYP genes was significantly modulated after caffeine exposure, we tested the effect of metyrapone, a general CYP inhibitor [35]. Male flies fed for 2 hours with the “caffeine + metyrapone” mixture produced much less caffeine metabolites in their body than controls (Fig. 1C). Furthermore, we manipulated the expression of several CYP candidate genes by using the GAL4/UAS-RNAi binary system. In particular, a ubiquitously expressed driver (*Actin*-GAL4), allowed us to separately target the RNAi of three CYP candidates (ds*CYP12d1*, ds*CYP6a8* and ds*CYP6d5*) that were highly up-regulated after caffeine exposure (Table 1). Given the simultaneous up-regulation of these three CYP genes by caffeine, we also measured their potential interaction and coordinated regulation.

**Fig 2:**
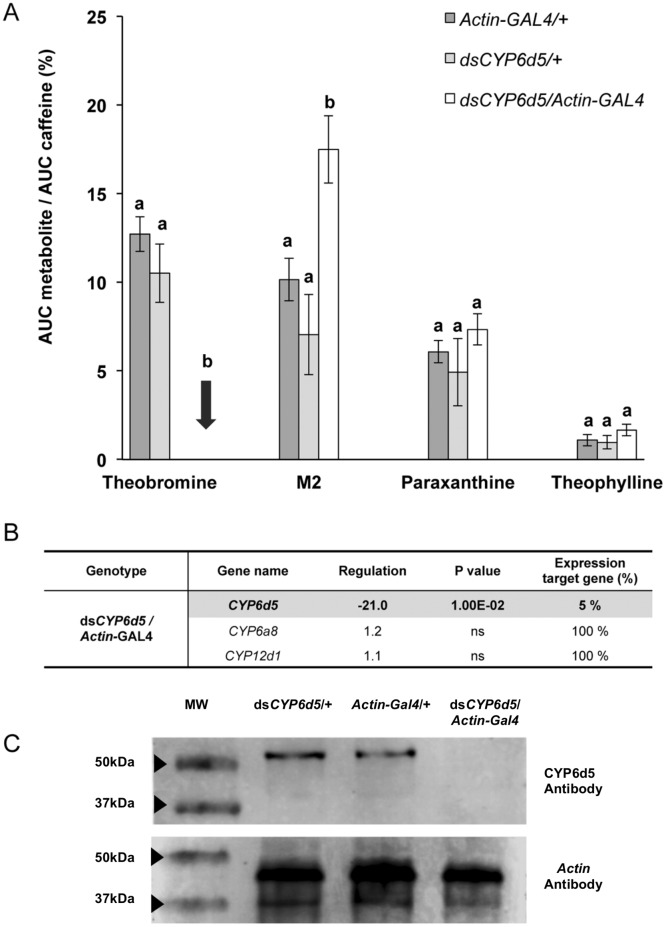
Effect of *CYP6d5* knockdown in Drosophila caffeine metabolism. A- The comparison of the normalized quantities of caffeine metabolites reveals a dramatic decrease of theobromine combined with a substantial increase of M2 in *CYP6d5* silenced flies (ds*CYP6d5*/*Actin*-GAL4) compared to the two transgenic parental controls (*Actin*-GAL4/+, ds*CYP6d5*/+). This analysis was performed by measuring the relative intensity of the radiolabelled signal detected in the bodies of male flies of these three genotypes. Bars represent mean values (±s.e.m). For each metabolite, the statistical differences are indicated by different letters (ANOVA, n = 4). B- CYP genetic targeting induces specific effect on transcript levels. The comparison of fold change expression between *CYP12d1*, *CYP6a8* and *CYP6d5* (measured with RT-qPCR) reveals that only *CYP6d5* level was affected in silenced *CYP6d5* males (ds*CYP6d5*/*Actin*-GAL4) (Statistical analysis by REST, p < 0.01; n = 3). Data are given relatively to normalized expression fold variation compared to controls. C- Comparison of expression level of CYP6D5 protein in experimental and control genotypes by western blotting with a CYP6D5 antibody. The Actin antibody was used to provide a control measurement.

First, ubiquitous silencing of *CYP6d5* strongly altered the production of two caffeine-derived metabolites in caffeine-exposed male flies: the production of theobromine was dramatically decreased, whereas M2 was significantly increased (in ds*CYP6d5/Actin*-GAL4). This effect was specifically due to *CYP6d5* silencing because no such variation was detected in the two transgenic controls (*Actin*-GAL4/+ and ds*CYP6d*5/+). Fig. 2A shows that the paraxanthine and theophylline levels did not differ between the RNAi-targeted and control strains. The silencing efficiency of ds*CYP6d5*, measured by RT-qPCR in the body of male adult flies, was measured not only on the expression of *CYP6d5* but also of *CYP12d1* and *CYP6a8* genes (Fig. 2B). The mRNA level of *CYP6d5* was strongly decreased (-95%) in ds*CYP6d5*/*Actin*-GAL4 strain, whereas the expression level of two other CYPs remained unaffected. Moreover, the level of the CYP6D5 protein was strongly decreased in ds*CYP6d5*/*Actin*-GAL4 males compared to transgenic control males as demonstrated by western blot (Fig. 2C).

The ubiquitous silencing effects of *CYP12d1* and *CYP6a8* on metabolism and transcription were also similarly assessed.

**Fig 3:**
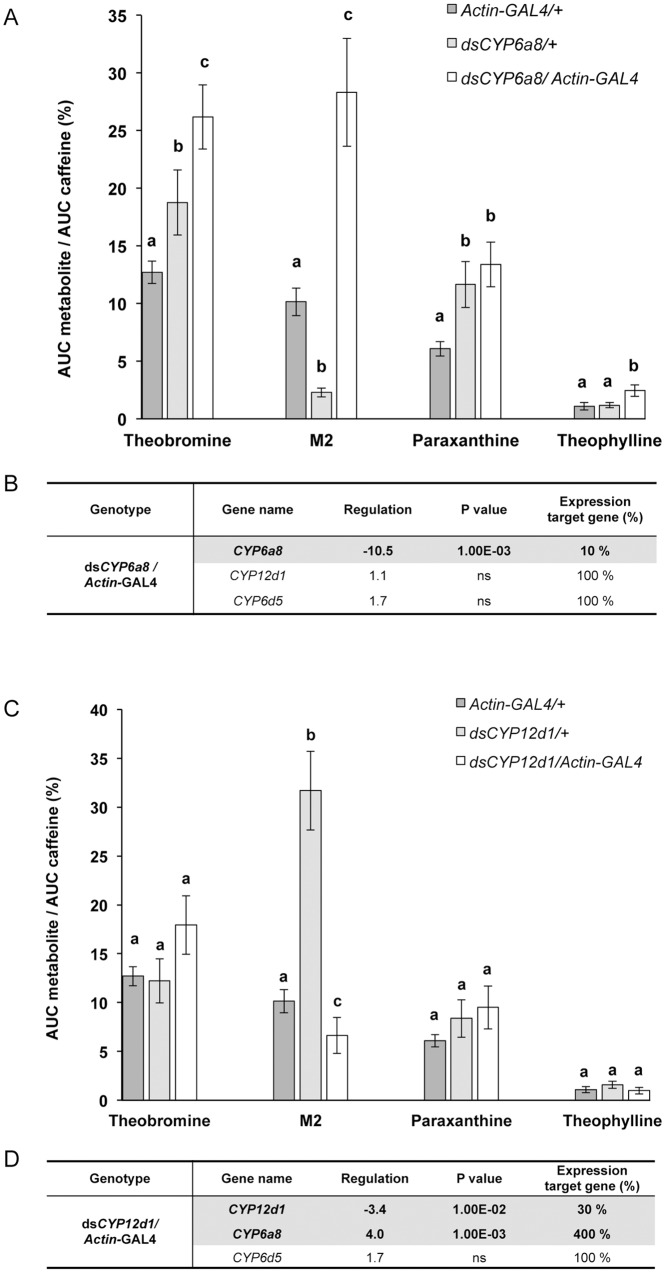
Influence of *CYP6a8* and *CYP12d1* genetic silencing in the metabolism of caffeine and gene expression. A and C: Comparison of normalized quantity of caffeine metabolites in males with respective silencing of *CYP6a8* and *CYP12d1* genes. The relative amount of metabolites was evaluated based on the intensity of the radiolabelled signal detected in the male bodies of experimental genotypes (ds*CYP6a8*/+ and ds*CYP12d1*/+ combined with *Actin*-GAL4/+) and transgenic controls. Bars represent mean values (±s.e.m.). For each metabolite, the statistical differences are indicated by different letters (ANOVA, n = 3). B and D: Comparison of fold change expression of *CYP12d1*, *CYP6a8* and *CYP6d5* in ds*CYP6a8*/*Actin*-GAL4 and ds*CYP12d1*/*Actin*-GAL4, respectively, and their transgenic controls. The quantitative variation of transcript level was measured with RT-qPCR analysis. Data are shown as normalized expression fold variation compared to controls. Highlighted data indicate statistical differences (p < 0.01; n = 4). For detailed methods and statistics, please refer to legend of Fig. 2.

Caffeine-exposed ds*CYP6a8*/*Actin*-GAL4 males produced much more theobromine, M2 and theophylline compared to transgenic controls (*Actin*-GAL4/+, ds*CYP6a8*/*+;* Fig. 3A). In ds*CYP6a8*/*Actin*-GAL4 flies, the mRNA level of *CYP6a8* was significantly decreased (-90%), whereas the transcription of *CYP12d1* and *CYP6d5* was not affected (Fig. 3B).

ds*CYP12d1*/*Actin*-GAL4 males fed with caffeine only showed a significant decrease of the M2 metabolite compared to controls (*Actin*-GAL4/+, ds*CYP12d1*/+; Fig. 3C). Moreover, if the mRNA level of *CYP12d1* was significantly decreased in ds*CYP12d1*/*Actin*-GAL4 males (-70%), the expression level of *CYP6a8* was strongly increased (+400%), whereas *CYP6d5* expression remained affected (Fig. 3D).

## Discussion

While the metabolism of caffeine has been extensively investigated in vertebrates, this is not the case in invertebrates. Medical and pharmacological applications based on human caffeine metabolism are widely available. For example, the measure of caffeine metabolites in urine provides an accurate assessment of an individual’s ability to metabolize drugs [43]. This measure is often based on the activity of CYP1A2, one of the main human enzymes involved in caffeine metabolism. In cases of combined ingestion of caffeine with pharmaceutical compounds, the measurement of CYP1A2 activity allows for precise adjustment of the optimal drug amount required for each person [44,45].

Despite the multiple effects induced by caffeine in insects (genome defect, growth, metamorphosis, sleep, pesticide adaptation, gene regulation), CYP-related metabolism of caffeine was previously unknown in invertebrates. We detected (on TLC) theobromine, paraxanthine and theophylline, three Drosophila caffeine-derived metabolites that are also present in mammals. This indicates that the substances resulting from caffeine metabolism have been partially conserved across evolution. We were not able to relate the 1, 3, 7-trimethyluric acid (TMUA) standard to any of the five unknown metabolites detected. Alternatively, the absence of 1, 3, 7- TMUA in flies can also be due to a slight change of the migratory properties (on TLC) of some caffeine metabolites induced by radiolabelling [46]. This “human *vs* Drosophila” variation for caffeine metabolites is not surprising, given the known inter-mammal differences. The origin of species-specific differences may be related to the main oxidation pathways that diverges between human (3-N demethylation), monkeys (7-N demethylation) and rat, mouse and rabbit (C-8 hydroxylation; [26]). Our data suggest that 1-N demethylation is the main Drosophila pathway leading to the production of 42% theobromine, whereas the secondary “3-N and 7-N demethylation” pathways lead to 20% paraxanthine and 4% theophylline, respectively.

Our pharmacological and genetic manipulation of Drosophila caffeine metabolism strongly supports the involvement of CYPs in this process. First, caffeine metabolism was drastically reduced in flies treated with metyrapone, a potent pharmacological inhibitor of CYP activity known to affect insect CYP in relation to hormone synthesis [47] and pheromone catabolism [35,48].

Second, genetic down-regulation of the expression of three CYP candidates revealed (i) CYP-specific effect on metabolism and (ii) CYP-CYP interactive regulation.

**Fig 4:**
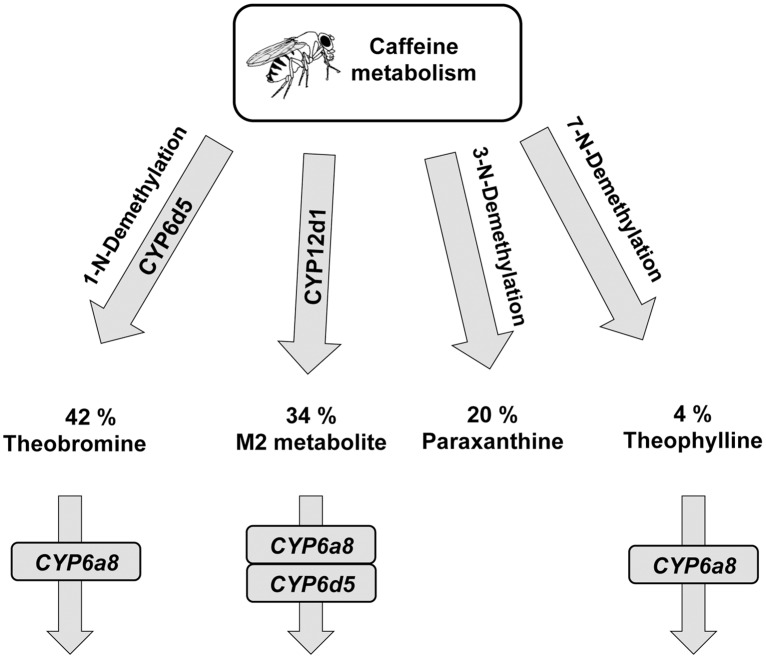
Hypothetic model for *CYP6d5*, *CYP6a8* and *CYP12d1* involvement in caffeine metabolism pathway in *Drosophila melanogaster*. In male adult fly bodies, caffeine is degraded into 4 major metabolites: theobromine, M2, paraxanthine and theophylline. *CYP6d5* and *CYP12d1* seem to metabolize caffeine in theobromine and M2 metabolite, respectively. The metabolism of theobromine and theophylline into unknown secondary metabolites may be controlled by *CYP6a8* whereas the M2 degradation could involve cooperation between *CYP6a8* and *CYP6d5*.

More precisely, genetic silencing of *CYP6d5* pinpoints its key-role in the synthesis of theobromine, but not in the two other identified metabolites. In contrast, *CYP6a8* or *CYP12d1* silencing induced a reciprocal effect on the level of theobromine that increased along with other metabolites. This indicates that *CYP6d5* and the two latter enzymes act in distinct steps of the caffeine metabolic pathway. Based on these results, we propose a hypothetical caffeine metabolism pathway in Drosophila (Fig. 4). Currently, we do not know whether another CYP acts with *CYP6d5*, as in humans with CYP1A2 [25]. Our genetic manipulation of the three CYPs indicates a possible regulatory interaction between these genes. If the manipulation of either *CYP6d5* or *CYP6a8* did not affect the expression of the two other tested enzymes, that of *CYP12d1* induced a 4-fold transcriptional increase in of *CYP6a8*, suggesting a potential mechanism of transcriptional compensation between these two CYPs (and possibly with other CYPs not studied here). The non-optimal efficiency of ds*CYP12d1* and the transcriptional compensation between *CYP12d1* and *CYP6a8* do not allow us to conclude that *CYP12d1* is directly implicated in the metabolic transformation of caffeine into M2 metabolite.

The regulation of genes expression by xenobiotic treatments or exposure is a universal feature of animals. Toxicity can result from genetic deregulation, either induced by the down-regulation of detoxification enzymes and/or by the up-regulation of enzymes involved in the bioactivation of xenobiotic compounds. Our transcriptomic screen allowed us to detect a large set of proteins potentially involved in caffeine catabolism. This includes phase I enzymes (CYPs), which often exhibited the highest response to the treatment, as well as phase II (GST and UGT) and phase III (ABC transporter) proteins. Because these proteins control xenobiotic detoxification, regulatory mechanisms may strongly enhance the kinetics of xenobiotic catabolism. Moreover, Drosophila xenobiotic response can be regulated by CncC (Cap n’ collar isoform C), a transcription factor ortholog to Nrf2 (NF-E2-related factor 2), which is activated by coffee [49] in humans [34]. Nine of the eleven CYP genes induced by caffeine in our study (*CYP12d1-p* and -*d*, *CYP6a8*, *CYP6d5*, *CYP4p1*, *CYP28a5*, *CYP12a5*, *CYP 6a20*, *CYP6w1*) were also up-regulated in transgenic flies over-expressing CncC [34]. Similarly, *CYP6a18*, *CYP313a1* and *CYP4d21* were down-regulated in CncC transgenic caffeine-fed flies. Furthermore, caffeine-induced effects depend on one binding site of CncC located in the promoter region of *CYP6a8* [34]. This strongly suggests that CncC could be a major player in the coordinated response to caffeine.

In addition to the fundamental relevance of our study, the response of CYPs to environmental stress may also represent a reliable marker with regard to the acquisition of resistance in insects repeatedly exposed to xenobiotics or to toxic compounds. One way to assess insect adaptation (and resistance) to caffeine and to other environmentally stressful molecules may be provided by the measure of theobromine amount (or other metabolites) after exposure to these toxic substances. Additionally, as previously successfully demonstrated with other CYPs, *CYP6d5* RNAi silencing could be used to screen for xenobiotic resistance [31]. Furthermore, given that CYPs are also involved in drug metabolism of vertebrates, the measurement of theobromine in Drosophila could offer a suitable tool for therapeutic drug discovery [50] and provide a simple but robust marker of CYP6D5 activity. For example, *CYP6d5* expression is up-regulated by the drug phenobarbital (barbituric compound), but the involvement of this enzyme in drug metabolism is not completely understood [51,52]. In parallel to drug response, CYP activity could explain the variation in response of Drosophila between mutants or natural populations, hence the potential adaptive response of their metabolism after caffeine exposure.

We have not studied the potential effect of metabolites—in particular of theobromine—in Drosophila because this metabolite was shown to induce a weaker effect than caffeine on mortality, female fecundity, and male mating preference [54]. Similar to humans, caffeine and theobromine were shown to differentially affect mood, psychomotor performance and blood pressure [53].

In summary, using Drosophila, we (i) discovered several products derived from caffeine metabolism, (ii) partly unrevealed the potential genetic network underlying this process, (iii) showed similarities between insects and mammals and (iv) demonstrated a high specificity of CYP6D5 with regard to caffeine degradation. With the current possibility to link the metabolic transformation of a natural compound with the transcriptomic identity of each animal, our study provides a useful base to design accurate bioassays for the evaluation of metabolic ability among individuals or between populations.
